# Persistent benefit of pharmacogenomic testing on initial remission and response rates in patients with major depressive disorder

**DOI:** 10.3389/fphar.2025.1658616

**Published:** 2025-10-30

**Authors:** Daniel Hain, Andria L. Del Tredici, Ryan B. Griggs, Rebecca Law, Brent Mabey, Holly L. Johnson, Katherine Johansen Taber, Kevin G. Lynch, Alexander Gutin, David W. Oslin

**Affiliations:** ^1^ Myriad Genetics, Salt Lake City, UT, United States; ^2^ Mental Illness Research, Education and Clinical Center, Corporal Michael J Crescenz VA Medical Center, Philadelphia, PA, United States; ^3^ Department of Psychiatry, University of Pennsylvania, Philadelphia, PA, United States

**Keywords:** pharmacogenetics, drug response, antidepressive agents, precision medicine, depression, pharmacogenomics

## Abstract

**Background:**

In patients with major depressive disorder (MDD), achieving remission and/or response may take many months because of the lengthy trial-and-error process often needed to identify effective medication. Pharmacogenomic testing is a prescribing tool that has been shown to improve remission and response rates for MDD patients, but data describing its impact over time is limited. The objective of this study was to determine whether pharmacogenomic-guided treatment increases the rate of remission and response over time in patients with MDD, and if so, to assess the persistence of that effect.

**Methods:**

This study was a prespecified *post hoc* analysis of the PRIME Care (Precision Medicine in Mental Healthcare) randomized clinical trial, a pragmatic trial that compared pharmacogenomic-guided treatment with usual care among veterans with depression. Participants were recruited at 22 Department of Veterans Affairs medical centers by 676 clinicians and were randomized to the pharmacogenomic-guided arm or the usual care arm. Multivariate Cox proportional hazards models were used to estimate hazard ratios (HR) and 95% confidence intervals (CI) for associations between study arm (pharmacogenomic-guided treatment or usual care) and the first instance of response or remission as assessed by the Patient Health Questionnaire-9 (PHQ-9) scale.

**Results:**

1,764 (90.7%) of the 1,944 veterans who participated in the PRIME Care trial had sufficient visit data to be included in this analysis. Patients who received pharmacogenomic-guided treatment had higher rates of remission (HR [95% CI] = 1.27 [1.05, 1.53]; p = 0.015) and response (HR [95% CI] = 1.21 [1.05, 1.40]; p = 0.010) at any time relative to patients receiving usual care. Schoenfeld residuals tests were not statistically significant for remission (p = 0.931) or response (p = 0.112), providing no evidence that the benefit due to pharmacogenomic-guided treatment changed over the 24-week period.

**Conclusion:**

Pharmacogenomic-guided treatment led to faster initial remission and response in patients with MDD, and this benefit persisted over 6 months with no evidence of changing over time.

## 1 Introduction

Major depressive disorder (MDD) is a significant cause of morbidity, with a lifetime prevalence of 20% in the United States and 4% worldwide (Hasin et al., 2018; World Health Organization, 2023). Treatment of MDD often involves the use of psychotropic medications to achieve clinically significant response and ultimately symptom remission. However, fewer than 40% of individuals achieve remission after their first antidepressant medication, and the chances of remission diminish with each subsequent medication trial (Rush et al., 2006). Since antidepressants can take several weeks to show efficacy (Gelenberg, 2010; McQuaid et al., 2022), a patient may require multiple medication trials over the course of many months before reaching remission. Approximately half of MDD patients receive two or more different medications in the 3 years following diagnosis, while one-third receive three or more (Kern et al., 2020). This trial-and-error prescribing approach may prolong the burden of MDD for the patient and increase healthcare costs (Ionescu et al., 2015).

Pharmacogenomic (PGx) testing is a prescribing tool that can identify medications that may require dose adjustments, be less likely to work, or have a higher risk of side effects by analyzing genetic variants that impact medication pharmacokinetics or pharmacodynamics (Bousman et al., 2023a). Guidelines for the use of PGx test results in prescribing antidepressants are available (Hicks et al., 2017; Bousman et al., 2023b; Beunk et al., 2024; Lam et al., 2023), and FDA labeling for many antidepressants includes PGx information (FDA, 2022). Data from multiple randomized clinical trials have shown that PGx-guided treatment increases the overall proportion of patients achieving remission from MDD compared to usual care (Greden et al., 2019; Oslin et al., 2022). A meta-analysis of thirteen trials including 4,767 MDD patients concluded that PGx-guided treatment is associated with a 41% higher likelihood of remission compared to treatment as usual (Brown et al., 2022). Subsequent meta-analyses have replicated the observation of higher remission rates with PGx-guided treatment in MDD (Arnone et al., 2023; Bunka et al., 2023; Wang et al., 2023; Milosavljević et al., 2024; Santenna et al., 2024; Albers et al., 2025).

PGx testing may improve remission and response rates in MDD by reducing trial-and-error prescribing. However, evidence demonstrating the impact of PGx-guided treatment on time-to-remission and time-to-response, as well as persistence of such effects over time, is limited. The Genomics Used to Improve DEpression Decisions (GUIDED) trial found that likelihood of remission in the PGx-guided arm doubled from 8 weeks to 24 weeks, suggesting a persistent effect (Greden et al., 2019). However, comparison to the control arm was not possible in GUIDED after 8 weeks because clinicians could access PGx results for patients in the control arm at that time.

The largest PGx trial conducted for depression, the Precision Medicine in Mental Healthcare (PRIME Care) study, evaluated 1,944 veterans with MDD and compared outcomes for PGx-guided versus usual care (Oslin et al., 2022). Compared to GUIDED, the PRIME Care study included a larger cohort size and longer duration for the primary outcome (over 24 weeks) and assessed depression outcomes at more time points after randomization (4, 8, 12, 18, 24 weeks). PRIME Care met both of its prespecified primary outcomes: patients in the PGx-guided arm were less likely to be prescribed an antidepressant medication with a significant gene-drug interaction and were 28% more likely to achieve remission across the 24-week duration of the trial compared to patients in the usual care arm. Additional analyses in the PRIME Care study showed that the proportion of remitters was significantly higher in the PGx-guided arm at 8 and 12 weeks but was not significantly different at 18 and 24 weeks. Although no interaction between time and study arm was observed in the analysis, the study concluded that provision of PGx test results had a nonpersistent effect on symptom remission; however, direct analysis was not performed to verify this statement (Oslin et al., 2022). Herein, we directly tested the hypothesis that PGx testing leads to persistently higher rates of MDD remission and response over time by assessing the impact of PGx testing on initial time-to-remission and time-to-response in the PRIME Care trial.

## 2 Materials and methods

### 2.1 Design and patient data

This study was a *post hoc* analysis, utilizing a prespecified analysis plan, of the PRIME Care study. Briefly, the PRIME Care trial was a randomized pragmatic clinical trial in which patients with MDD and at least one treatment failure were randomized to receive either PGx-guided treatment or usual care for a period of 24 weeks at the beginning of an antidepressant treatment episode. Baseline assessments were performed prior to randomization, with post-randomization follow-up visits scheduled at 4, 8, 12, 18, and 24 weeks. A full description of the trial, including eligibility and exclusion criteria, is available (Oslin et al., 2021; Oslin et al., 2022). This *post hoc* study was reviewed by the Advarra Institutional Review Board and determined to qualify as exempt research per 45 CFR 46.104(d)(4).

Relevant clinical and demographic information collected at the baseline or follow-up visits included age, race (Black/African American, White, Other/unspecified), Hispanic ethnicity, sex (female, male), smoking status (cigarettes per day over the prior 30 days), patient-reported psychotropic treatment history, reported visit date (days since randomization), and practice location (primary care, mental healthcare, or integrated care) (Table 1).

**TABLE 1:** Baseline patient characteristics.

	Group, no. (%)		
Characteristic	Pharmacogenomic-guided (N = 884)	Usual care (N = 880)	p-value^a^
Patient characteristics			
Age			0.918
60+	241 (27)	237 (27)	
<60	643 (73)	643 (73)	
Sex			0.110
Female	205 (23)	234 (27)	
Male	679 (77)	646 (73)	
Race			0.291
Black	170 (19)	152 (17)	
White	588 (67)	616 (70)	
Other/unspecified	126 (14)	112 (13)	
Ethnicity			0.638
Hispanic	104 (12)	92 (10)	
Non-Hispanic	777 (88)	784 (89)	
Unspecified	3 (0)	4 (0)	
Smoking status			0.857
Smoker	151 (17)	158 (18)	
Non-smoker	727 (82)	717 (81)	
Unspecified	6 (1)	5 (1)	
Clinical symptoms			
PHQ-9 score mean (SD)	17.5 (4.3)	17.4 (4.3)	0.756
Treatment refractory^b^	270 (31)	273 (31)	0.868
PTSD presence	517 (58)	506 (58)	0.776
Practice location			0.853
Integrated care	163 (18)	162 (18)	
Primary care	109 (12)	101 (11)	
Specialty mental health	612 (69)	617 (70)	

^a^ To assess for differences between study arms, chi-squared tests were used for categorical variables and the Wilcoxon rank-sum test was used for baseline PHQ-9, score.

^b^ Treatment-refractory depression is defined as self-reported history of 2 or more medication treatments for at least 6 weeks with standard doses or treatment with electroconvulsive therapy or transcranial magnetic stimulation.

### 2.2 Clinical assessments and endpoints

Assessments relevant for this analysis, conducted at baseline and each follow-up visit, included the Patient Health Questionnaire-9 (PHQ-9) (Spitzer et al., 1999) and the Post Traumatic Stress Disorder (PTSD) Checklist for DSM-5 (PCL-5) (Weathers et al., 2013).

The primary endpoints of this analysis were the time of the first instance of remission (defined as PHQ-9 ≤5) and response (defined as ≥50% reduction from baseline PHQ-9 score). PHQ-9 scores range from 0–27 points.

### 2.3 Statistical analysis

Baseline characteristics were compared between study arms using chi-squared tests for categorical variables and Wilcoxon rank-sum test for baseline PHQ-9 score to assess significant differences, with a two-sided α level of 5%.

In the primary analysis, two separate multivariate Cox proportional hazards models (one for remission, one for response) were used to estimate hazard ratios (HRs) and 95% confidence intervals (CIs) for associations between study arm (PGx-guided or usual care) and the first instance of either PHQ-9 remission or PHQ-9 response, respectively, measured at each follow-up visit (Klein and Moeschberger, 2003). Baseline PHQ-9 score was included as the only additional covariate in each model as an explanatory variable. The Efron method was selected to handle tied event times. The statistical significance of individual explanatory variables was determined using likelihood ratio tests. The proportional hazard assumption for the study arm variable was assessed using the Schoenfeld residuals test. Two-sided tests of significance were used, with a Bonferroni-adjusted α level of 0.025 (0.05/2) to account for testing two outcomes. Cox models used study week (4, 8, 12, 18, or 24 weeks) as the time scale in accordance with the timing of data collection in the PRIME Care study design (set prior to this *post hoc* analysis). Patients were right-censored at the first instance of a missing follow-up visit (defined as any visit without an associated PHQ-9 score). Patients were also right-censored at the first instance of a follow-up visit occurring outside of the expected time window based on reported visit date (14–42 days [week 4], 42–70 days [week 8], 70–105 days [week 12], 105–147 days [week 18], 147–189 days [week 24]). These windows were defined *post hoc*, based on the midpoints between the weeks when follow-up visits were scheduled to occur. Patients were excluded from the analysis if they only had baseline data remaining after right-censoring (Supplementary Figure S1). As a sensitivity analysis, models were re-analyzed without the right-censoring based on reported visit date. As an additional sensitivity analysis, models were re-analyzed with the inclusion of additional prespecified covariates (age [60+], race, Hispanic ethnicity, sex, presence of PTSD at baseline, history of treatment refractory depression, smoking status [≥1 cigarette per day over the prior 30 days], and practice location).

All analyses were conducted using R software version 4.4.1 (R Foundation for Statistical Computing, 2021).

## 3 Results

### 3.1 Baseline characteristics

Out of the 1,944 patients in the PRIME Care study, 180 (9.3%) were excluded due to having only baseline data remaining after right censoring, leaving 1,764 patients available for analysis (90.7%; n = 884 in the PGx-guided arm and n = 880 in the usual care arm), with the majority being under 60 years of age (73% in the PGx-guided arm, 73% in the usual care arm) and male (77% in the PGx-guided arm, 73% in the usual care arm) (Table 1; Supplementary Figure S1). This cohort was also mostly White (67% in the PGx-guided arm, 70% in the usual care arm), non-Hispanic (88% in the PGx-guided arm, 89% in the usual care arm), and non-smoking (82% in the PGx-guided arm, 81% in the usual care arm). Both arms had equal proportions of individuals with moderately severe depression (mean PHQ-9 values of 17.5 and 17.4, respectively), treatment refractory depression (31%), and comorbid PTSD (58%).

Baseline patient characteristics were compared by study arm (Table 1). No statistically significant differences were observed across study arms for any baseline characteristic.

### 3.2 Time-to-event analysis

**FIGURE 1:**
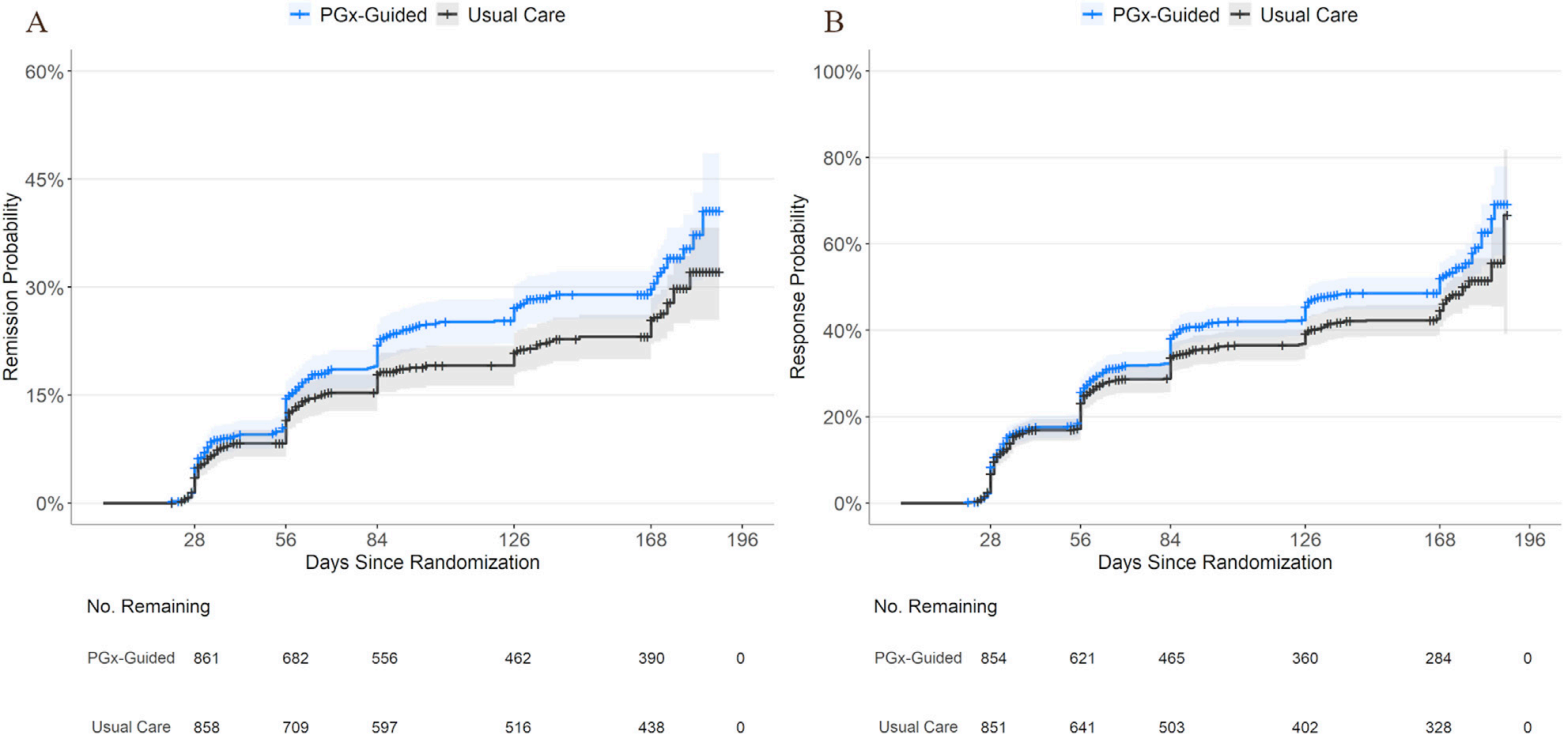
Cumulative Incidence of Remission and Response by Study Arm. Kaplan-Meier curves showing cumulative incidence of remission **(A)** and response **(B)** over time for patients receiving PGx-guided treatment or usual care using reported visit date (days since randomization) as the timescale for clarity. Shaded areas indicate 95% confidence intervals. The number of individuals remaining event-free per treatment group are shown below the x-axis. Censoring is indicated by cross tick marks on each curve. Note that the primary analysis used study week as the timescale and group differences were not evaluated using reported visit date.

Cox proportional hazards models and likelihood ratio tests revealed that patients who received PGx-guided treatment had higher rates of remission (HR [95% CI] = 1.27 [1.05, 1.53]; p = 0.015) and response (HR [95% CI] = 1.21 [1.05, 1.40]; p = 0.010) at any time relative to patients receiving usual care. Kaplan-Meier plots, using reported visit date as the timescale, show that the cumulative incidence of remission and response appeared higher in the PGx-guided arm starting early during the study period and continued through the end of the study period (Figure 1).

**FIGURE 2:**
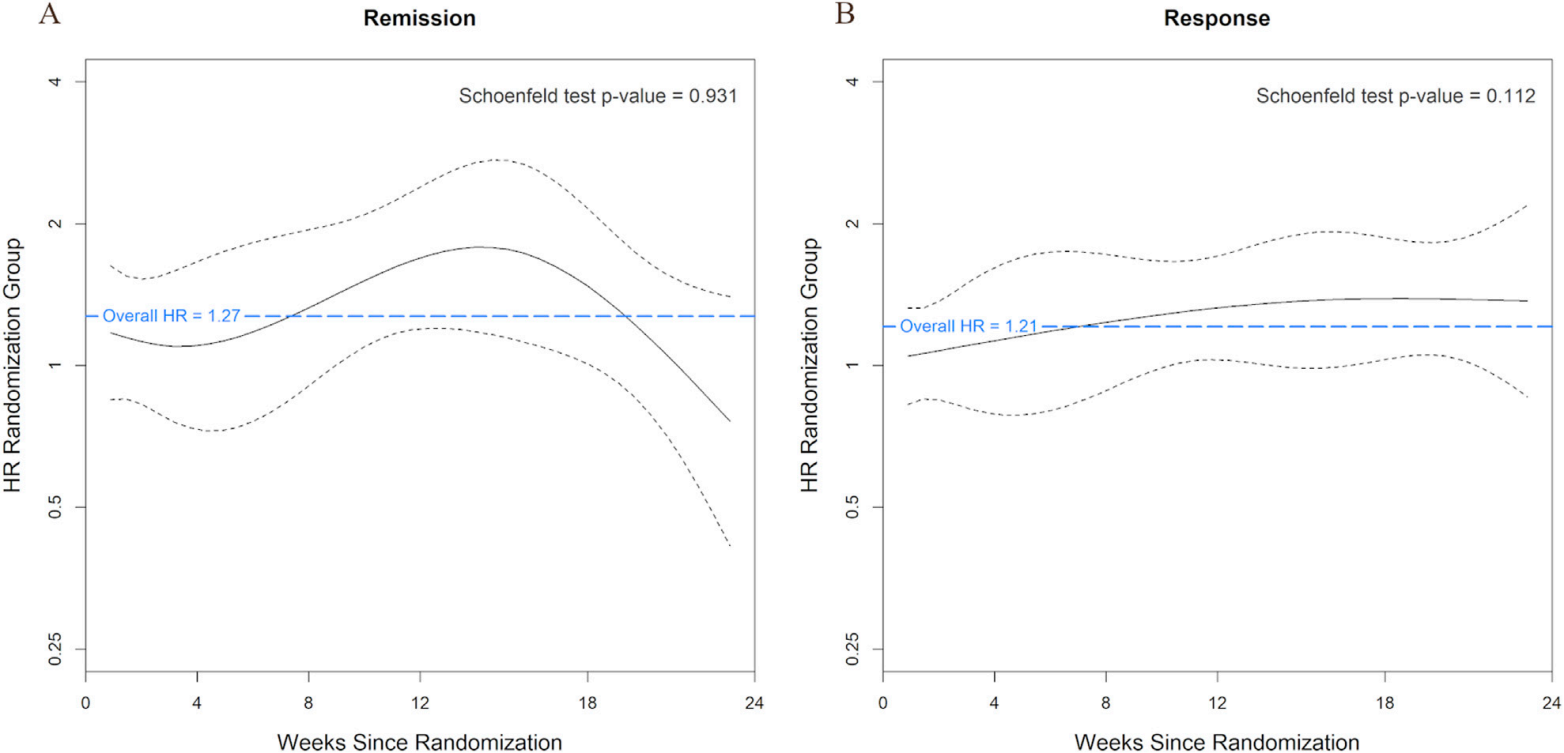
Time-Varying Effects of Study Arm on Remission and Response on the Log Hazard Ratio Scale. Smoothed trend lines of scaled Schoenfeld residuals for the effect of study arm on remission **(A)** and response **(B)** using study week as the timescale. A blue horizontal dashed line indicates the overall hazard ratio estimated from the Cox model. Black dashed lines represent the 95% confidence band around the smoothed effect estimate over time. Systematic deviation from the confidence band may indicate violation of the proportional hazards assumption.

The Schoenfeld residuals test of the study arm variable was not statistically significant for remission (p = 0.931) or response (p = 0.112), indicating no evidence of time-dependent effects of study arm, consistent with the proportional-hazards assumption. Schoenfeld residual plots did not show any significant fluctuation of the remission or response hazard ratios over time (Figure 2). These results indicate that the benefit due to PGx-guided treatment did not change significantly over the 24-week period.

### 3.3 Sensitivity analyses

As a sensitivity analysis, models were re-analyzed without right-censoring based on reported visit date, resulting in fewer (n = 125 [6.4%]) patients excluded. Among patients included in the sensitivity analysis (n = 1819 [93.6%]), the associations between study arm and time-to-remission (HR [95% CI] = 1.24 [1.03, 1.49]; p = 0.022) or response (HR [95% CI] = 1.20 [1.05, 1.38]; p = 0.010), as well as Schoenfeld residuals test results for study arm and remission (p = 0.699) or response (p = 0.227), were similar to those seen in the primary analysis. Models were also re-analyzed with the inclusion of additional prespecified covariates (age [60+], race, Hispanic ethnicity, sex, presence of PTSD at baseline, history of treatment refractory depression, smoking status [≥1 cigarette per day over the prior 30 days], and practice location). After adjusting for these prespecified covariates, associations between study arm and time-to-remission (HR [95% CI] = 1.30 [1.07, 1.57]; p = 0.008) or response (HR [95% CI] = 1.23 [1.07, 1.43]; p = 0.004), as well as Schoenfeld residuals test results for study arm and remission (p = 0.990) or response (p = 0.092), were similar to those seen in the primary analysis. Primary analysis findings were robust to changes in right-censoring and to the inclusion of additional covariates.

## 4 Discussion

This is the first study to demonstrate that for patients with MDD, PGx-guided treatment is associated with higher rates of initial remission and response that persist for 6 months after PGx testing, with no evidence of the effect changing over time. These findings build upon previous studies showing that PGx testing increases the overall proportion of MDD patients achieving remission and response compared to usual care (Greden et al., 2019; Brown et al., 2022; Oslin et al., 2022; Arnone et al., 2023; Bunka et al., 2023; Wang et al., 2023; Milosavljević et al., 2024; Santenna et al., 2024).

Time-to-event analyses in this study indicated that PGx-guided treatment was associated with a 27% higher rate of remission and a 21% higher rate of response at any time between 4–24 weeks after randomization compared to usual care. The original PRIME Care analysis found that the overall proportion of remitters was not significantly different at the 18 weeks (16% in the PGx-guided arm, 14% in the usual care arm) or 24 weeks (17% in PGx-guided arm, 16% in the usual care arm) time points (Oslin et al., 2022). However, in the original analysis, all remission and response events were analyzed, while in this study, only the first remission or response event after randomization was evaluated. As such, the original analysis (Oslin et al., 2022) may have included patients who had relapsed and had a subsequent response or remission. Relapse, defined as a return of depressive symptoms, is a common event affecting about one in five MDD patients within a year of achieving remission (Geddes et al., 2003), and is likely to have occurred in the dataset. The higher rates of remission and response observed in this study indicate that patients in the PGx-guided arm achieved remission and response faster than those in the usual care arm. These findings are consistent with a recent study showing that PGx-guided dosing of tricyclic antidepressants led to faster attainment of therapeutic plasma concentrations compared to usual treatment (Vos et al., 2023). Achieving remission sooner in a patient’s treatment trajectory may have long-term clinical benefits. Analyses of the Sequenced Treatment Alternatives to Relieve Depression (STAR*D) trial (Rush et al., 2006) have shown that patients with earlier remission had both lower rates of relapse and a longer time to relapse in the year following remission (Rush et al., 2006; Kubo et al., 2023). Similarly, in another study, those who achieved response or remission within the first 6 weeks of antidepressant treatment were more likely than later remitters to still be in remission at 1 year after starting treatment (Ciudad et al., 2012). Additional clinical benefits of faster remission and response may include reduced risk of treatment-resistant depression (Nie et al., 2018; Arnaud et al., 2023). Furthermore, faster remission may also reduce residual symptoms, such as reactivity of mood, feeling sad, and pleasure/enjoyment. In a study of 1,595 depressed patients, those who achieved remission after 6–8 weeks of treatment had fewer and less severe residual symptoms compared to those who achieved remission after 16–20 weeks of treatment (Roca et al., 2011).

Faster remission and response may also have economic benefits. In the United States, the total economic burden of MDD has been estimated at $333.7 billion in 2019, including direct healthcare costs as well as indirect work-related costs (e.g., unemployment, productivity loss, absenteeism) (Greenberg et al., 2023). In one simulation, a novel therapy with faster time-to-response had an estimated cost savings of $25 billion per year (Greenberg et al., 2023). Reduction in relapse and incidence of treatment-resistant depression, driven by faster remission, is also predicted to reduce healthcare costs (Gauthier et al., 2019; Li et al., 2020; Touya et al., 2022).

The persistent impact of PGx on remission and response rates over time demonstrated in this study may explain new findings that PGx-guided care reduces healthcare resource utilization, including psychiatric hospitalizations (Del Tredici et al., 2025). Indeed, we hypothesize that the benefit of PGx-guided treatment may well extend beyond the 6-month timeframe of this study. Sustained remission is the ultimate treatment goal for patients with MDD (Trivedi and Daly, 2008), and PGx-guided treatment with conventional antidepressants could be more effective over time than newer rapidly acting agents, whose durability is unproven (Schatzberg and Mathew, 2024).

### 4.1 Strengths and limitations

The PRIME Care study, on which this analysis was based, was conducted on a large cohort of depressed patients and the design was pragmatic to represent real-world clinical practice (Oslin et al., 2022). The PRIME Care study also followed a prespecified analysis plan, and included multiple time points to assess depression outcomes between the PGx-guided and usual care arms over 24 weeks after randomization (Oslin et al., 2022). Building upon the original study, which assessed outcome differences at each time point individually, the current analysis was able to directly assess timing of initial response and remission across the entire study duration. Moreover, this analysis expands upon the non-significant interaction between arm and time reported in Oslin et al. (2022) by using statistical methods that directly assess time-to-event, providing a more statistically principled and clinically interpretable framework for evaluating the persistence of study arm effects.

While the PRIME Care study was the largest randomized clinical trial evaluating depression outcomes with and without PGx testing, the study design did not include time points after 24 weeks. As such, this analysis could not evaluate the persistence of the impact of PGx-guided treatment for longer than 24 weeks. Moreover, only time-to-first response or remission was evaluated, and any response/remission events occurring after relapse were not included in the analysis. Additionally, the outcomes observed in this study were limited to a specific PGx test and may not apply to all PGx tests. Finally, this study was conducted in a cohort of veterans, which may not be representative of the MDD patient population in the United States or worldwide; for example, the frequencies of PTSD and male sex were higher than in other large US depression cohorts.

## 5 Conclusion

Pharmacogenomic testing led to faster initial remission and response in patients with MDD. This effect persisted over 6 months without any evidence of changing over time. Future directions may include evaluating the economic impact of higher remission and response rates due to PGx testing, studying the effect of PGx testing on relapse, and analyzing other depression cohorts to understand the generalizability of these findings.

## Data Availability

Publicly available datasets were analyzed in this study. This data can be found here: Deidentified participant data is available. Contact dave.oslin@va.gov to propose secondary study analyses. If proposal is approved, a data dictionary and supporting documentation will be available via file transfer.

## Supplementary material

SUPPLEMENTARY FIGURE S1 Patient Flow Diagram. ^1^A *post hoc* sensitivity analysis was performed including these patients. Flow diagram showing which patients were excluded from analysis due to missing the initial follow-up visit (week 4) or having the initial follow-up visit occur outside the expected timeframe.
