# Supplementary figures and images for: Persistent benefit of pharmacogenomic testing on initial remission and response rates in patients with major depressive disorder
Source: Front Pharmacol. 2025 Oct 30;16:1658616. doi: 10.3389/fphar.2025.1658616 (PMC12611804; doi:10.3389/fphar.2025.1658616)

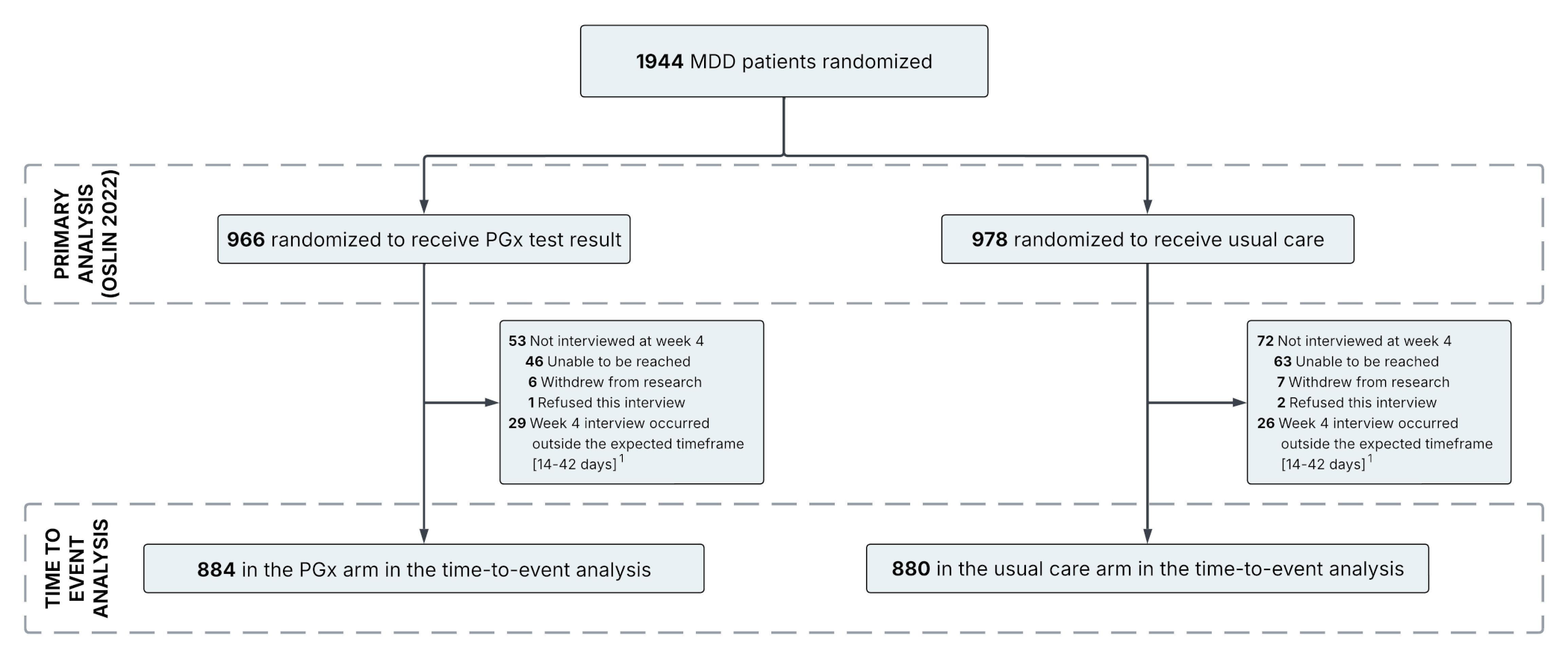

Supplement: Supplementary file 1 [file Image1.tiff]
